# Low dose radiation induced senescence of human mesenchymal stromal cells and impaired the autophagy process

**DOI:** 10.18632/oncotarget.2692

**Published:** 2014-12-16

**Authors:** Nicola Alessio, Stefania Del Gaudio, Stefania Capasso, Giovanni Di Bernardo, Salvatore Cappabianca, Marilena Cipollaro, Gianfranco Peluso, Umberto Galderisi

**Affiliations:** ^1^ Sbarro Institute for Cancer Research and Molecular Medicine, Center for Biotechnology, Temple University, Philadelphia, PA 19107-6799, USA; ^2^ Department of Experimental Medicine, Biotechnology and Molecular Biology Section, Second University of Naples, Naples 80138, Italy; ^3^ Institute of Bioscience and Bioresources, CNR, Naples 80138, Italy; ^4^ Department “F. Magrassi – A. Lanzara” Second University of Naples, Naples 80138, Italy

**Keywords:** Mesenchymal stem cells, senescence, radiation

## Abstract

Low doses of radiation may have profound effects on cellular function. Individuals may be exposed to low doses of radiation either intentionally for medical purposes or accidentally, such as those exposed to radiological terrorism or those who live near illegal radioactive waste dumpsites.

We studied the effects of low dose radiation on human bone marrow mesenchymal stromal cells (MSC), which contain a subpopulation of stem cells able to differentiate in bone, cartilage, and fat; support hematopoiesis; and contribute to body's homeostasis.

The main outcome of low radiation exposure, besides reduction of cell cycling, is the triggering of senescence, while the contribution to apoptosis is minimal. We also showed that low radiation affected the autophagic flux. We hypothesize that the autophagy prevented radiation deteriorative processes, and its decline contributed to senescence.

An increase in ATM staining one and six hours post-irradiation and return to basal level at 48 hours, along with persistent gamma-H2AX staining, indicated that MSC properly activated the DNA repair signaling, though some damages remained unrepaired, mainly in non-cycling cells. This suggested that the impaired DNA repair capacity of irradiated MSC seemed mainly related to the reduced activity of a non-homologous end-joining (NHEJ) system rather than HR (homologous recombination).

## INTRODUCTION

In adulthood, tissue-specific stem cells regulate homeostatic tissue regeneration. Stem cells are located in specific areas of tissues, called niches, and are characterized as being in a state of relative proliferative quiescence, from which they can exit under the proper conditions to obtain the proliferative potential necessary for tissue regeneration [1].

Stem cells reside for long periods of time in our bodies, and this increases the possibility that they may be subjected to genotoxic damage, which may derive from extrinsic (ionizing radiation, drugs, chemicals, etc.) or intrinsic sources (DNA replication errors, spontaneous chemical changes to DNA, programmed DNA recombination). Following damage, cells may properly repair DNA and restore functionality, or they may accumulate irreversible damages that trigger either apoptosis or senescence. Alternatively, damaged cells may undergo transformation with the onset of cancer [1, 2].

It is well known that high doses of ionizing radiation (IR), either as a side effect of therapeutic treatment or through accidental exposure to environmental sources, have a strong genotoxic effect. Normal tissues are extremely vulnerable to the cytotoxicity caused by high doses of IR. For example, IR may alter the functionality of the bone marrow and affect the resident stem cells. Several studies on the effects of IR have focused on its negative influence on hematopoietic stem cells (HSC), while only a few have addressed the effect of IR on the other cell types present in bone marrow, such as mesenchymal stem cells. These cells are of great interest since they differentiate in bone, cartilage, and fat; support hematopoiesis; contribute to the homeostatic maintenance of many organs and tissues; and modulate inflammatory response [3–9].

In recent years, there has been growing evidence that even low doses of radiation (< 500 mGy) may have profound effects on cellular functions. People may be exposed to low dose IR for medical purposes. The effects of low dose radiation related to medical imaging procedures are often not adequately monitored, although these procedures can be frequently repeated on the same individual. A recent study carried out on almost 1,000,000 non-elderly adults in healthcare markets across the United States showed that a consistent number of patients received up to 50 mGy/year [10, 11].

People may be also unintentionally exposed to low dose IR, such as those who are frequent flyers, those exposed to radiological terrorism and those who live near illegal IR waste dumpsites. For example, in our region, in Southern Italy, years of illegal practices of waste dumping increased risks for liver and lung cancer [12, 13].

The major target of low dose effects may be stem cells. As result of their long life, stem cells may undergo several rounds of low-level radiation damage that, taken singly, may not have a big impact on cellular physiology, but collectively, these rounds of radiation may severely affect cellular function.

We decided to analyze the effects of low dose radiation on human bone marrow mesenchymal stromal cells (MSC), which contain a subpopulation of stem cells, since to our knowledge there are only a few papers that partially address this topic in spite of the key role of MSC in bone marrow physiology [14, 15]. We explored whether a low dose of radiation affects the biology of MSC by inducing cytotoxic effects. In detail, we analyzed the process that cells use to counteract the negative effects of IR. Depending on radiation level and cell type, IR induces cell death by apoptosis, or does not kill cells but triggers senescence. We also evaluated if low IR may alter the autophagic flux. This last issue is of great interest since autophagy has opposing functions in response to IR stress. One is a cytoprotective function by the elimination of IR-damaged cellular components; the other is a cytotoxic effect that by destroying vital components may function to promote cell death [16].

## RESULTS

### MSC were sensitive to very low dose radiation

Several reports have clearly demonstrated that radiation survival of mammalian cells is a complex phenomenon, since it does not follow a simple linear dose/effect response. Many cell types show hyper-radiosensitivity (HRR) to very low levels of radiation (< 100 mGy) that cannot be predicted by extrapolating the cell survival response from high doses. There is also evidence for a radiation-induced protective response (increased radioresistance, IRR) at doses higher than 100/300 mGy and lower than 600/1000 mGy. Several cells may exhibit both HRR and IRR, or only one of the two phenomena [17–19].

As a preliminary study, we decided to investigate whether HRR and/or IRR could be present in MSC cultures. To this end, we irradiated MSC cultures with four different doses of X-rays (40, 160, 640 and 2000 mGy), and six hours post-irradiation we detected effects on cell cycle, apoptosis and senescence (Tab. 1). Irradiated MSC showed a reduction of cycling cells (Ki67+). This was already evident at the lowest dose we used, and we did not see a progressive increase with the highest doses. A similar trend was observed for apoptosis: irradiated cells showed a significant increase in the percentage of apoptotic cells compared with the control, but this did not grow progressively as the doses were increased (Tab. 1). On the other hand, the number of senescent cells rose as the X-ray dose increased (Tab. 1).

**Table 1:** Radiosensitivity of MSC The table shows the percentage of cycling (Ki67+), apoptotic and senescent cells six hours after X-ray treatment (40, 160, 640 and 2000 mGy). Table reports also clonogenic property of MSC, that was evaluated 15 days post-irradiation with CFU assay. Data are expressed with standard deviation (*n* = 3, *p < 0.05, **p < 0.01).

	Ki67+	Apoptotic	Senescent	CFU
0 mGy	28.8 ± 4.3%	3.6 ± 0.5%	16.8 ± 2.1%	360 ± 25
40 mGy	20.3 ± 2.8%	6.9 ± 0.9% *	25.6 ± 3.2% *	218 ± 10 *
160 mGy	23.6 ± 3.3%	5.2 ± 0.7% *	31.1 ± 4.3% *	206 ± 11 *
640 mGy	19.6 ± 3.5%	6.8 ± 0.8% *	36.3 ± 4.7%**	225 ± 13 *
2000 mGy	21.9 ± 3.7%	6.8 ± 0.9% *	40.0 ± 5.6% **	100 ± 4 **

The control of the stem cell properties (self-renewal, multipotentiality) is strictly linked to regulation of the cell cycle, control of apoptosis and senescence; hence, it is reasonable to have perturbation in stem cell status in irradiated MSC. We carried out a CFU assay on these cells to test their clonogenicity, i.e. their ability to expand at a single-cell level, which is an important feature of self-renewing stem cells. All the X-ray doses produced a reduction in the number of clones observed in 100 mm plates seeded at low density. In detail, the highest decrease was observed at 2000 mGy, while in the range 40–640 mGy the reduction of clonogenicity was constant. Together, these data suggest that MSC exhibit radiosensitivity to very low radiation dose (40 mGy), but as the dose level rises there is increased radioresistance until at doses below 2000 mGy. This is agreement with similar phenomena observed in other cell types [17–19].

### Low dose radiation reduced percentage of S-phase cells and promoted cell cycle exit

We decided to seek further insight into the effect of low dose radiation on the biology of MSC. To this end, we carried out our analysis by treating cells with the lowest dose we previously tested (40 mGy). Studies on cell sensitivity at very low doses are of extreme interest given the increasing number of persons that may be exposed to this dosage level and the possible clinical consequences [10, 11]. As reference, we studied the effects of a high dose (2000 mGy) on MSC functions.

**Figure 1:**
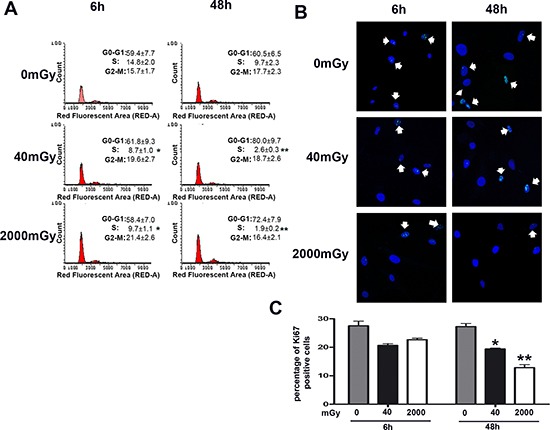
Cell cycle analysis of irradiated MSC Panel **(A)** – The picture shows representative FACS analysis of irradiated (40 and 2000 mGy) and control MSC. The experiments were carried out six and 48 hours post-irradiation. Experiments were conducted in triplicate for each condition. Percentage of different cell populations (G1, S and G2/M) are indicated. Data are expressed with standard deviation (*n* = 3, *p < 0.05, **p < 0.01). Panels **(B, C)** – Representative microscopic field of Ki67 immunostaining (green) on MSC six and 48 hours post-irradiation with 40 and 2000 mGy. Nuclei were counterstained with Hoechst 33342 (blue). Arrows indicate Ki67-positive cells. The graph shows the percentage of Ki67-positive cells. Data are expressed with standard deviation (*n* = 3, *p < 0.05, **p < 0.01).

The cell cycle analysis that we performed six and 48 hours post-irradiation showed that both low and high dose radiation induced a significant reduction of S-phase cells. In detail, 48 hours post-irradiation, we detected 2.6% and 1.9% cells in S-phase in 40 mGy and 2000 mGy treated cells, respectively, as compared to 9.7% in control cultures (Fig. 1A). These data are in good agreement with Ki67 immunostaining that evidenced a significant reduction of cycling cells in low dose irradiated cells compared with healthy cells (19.2% versus 27.1% 48 hours post-irradiation). The high-dose treatment generated a reduction in the number of cycling cells that was higher of that induced by low dose only at 48 hours (Fig. 1B, C). Concordantly, the proliferation rate of 40 and 2000 mGy treated cells was significantly lower than that of the control (Suppl. File 1).

### Low dose radiation induced senescence

**Figure 2:**
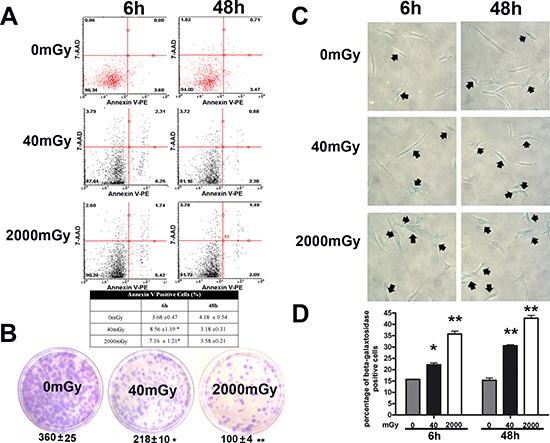
Analysis of senescence, apoptosis, stemness and autophagy in irradiated MSCs Panel **(A)** – Flow cytometry analysis of apoptosis with Nexin assay. The experiments were carried out six and 48 hours post-irradiation. The assay allows the identification of early (Annexin V + and 7ADD −) and late apoptosis (Annexin V + and 7ADD +). Nevertheless, apoptosis is a continuous process and we calculated the percentage of apoptosis as the sum of early and late apoptotic cells, to avoid a discretional separation between early and late apoptosis. The table shows the global percentage of Annexin V-positive cells. Data are expressed with standard deviation (*n* = 3, *p < 0.05). Panel **(B)** – CFU assay. The pictures show representative crystal violet staining of clones obtained after 14 days of incubation, with MSCs plated following irradiation experiments. The mean number of clones per 1,000 cells plated in 100 mm dish (± SD, *n* = 3, *p < 0.05, **p < 0.01) is indicated below each picture. Panels **(C, D)** – Senescence assay. Representative microscopic fields of acid beta-galactosidase (blue) in irradiated and control cells are shown. Arrows indicate senescent cells. The graph shows mean percentage value of senescent cells (± SD, *n* = 3, *p < 0.05).

We then analyzed the level of apoptosis and senescence by annexin V and acid-beta-galactosidase assay, respectively (Fig. 2). Six hours post treatment we detected an increase in apoptosis in both experimental conditions, but the apoptosis rate dropped below the control level at 48 hours (Fig. 2A). This suggests that apoptosis is an acute reaction to IR, while long-lasting effects may be associated to other phenomena. Indeed, a huge percentage of cells entered senescence six hours following IR, both for the low and high dose radiation (Fig. 2C, D). This percentage further increased at 48 hours. Senescence may be considered the “preferential answer” of MSC to stress induced by IR. This result is in good agreement with data on cell proliferation and clonogenic properties of MSC, as detected by quick proliferation assay and CFU evaluation, respectively (Suppl. File 1; Fig. 2B). In fact, senescence could greatly affect the “stemness” of MSC cultures.

### Autophagy process is impaired by low radiation

In the context of IR stress, the study of autophagy is of great interest since, depending on cellular type and quality and dose of radiation, autophagy may contribute to radioresistance or to increased sensitivity [16, 20–22]. Moreover, senescence and autophagy, which are closely related mechanisms that cells use to protect themselves from external and internal stress, have a complex relationship, since autophagy may promote or counteract senescence [23, 24].

We used the Vivadetect autoflux assay (VivaBioscience) to evaluate autophagy in IR-treated cells. The assays measured the levels of the microtubule-associated protein 1 light chain 3 (LC3), a reliable marker of autophagosome. It has two isoforms: LC3-I and LC3-II. We analyzed the autophagic flux by tracking the conversion of LC3-I proteins to LC3-II. Following synthesis, LC3 is processed by mammalian Atg4s and is present in the cytosol as LC3-I. When autophagy is induced, some LC3-I is converted into LC3-II, which is tightly bound to the autophagosome membrane [25].

**Figure 3:**
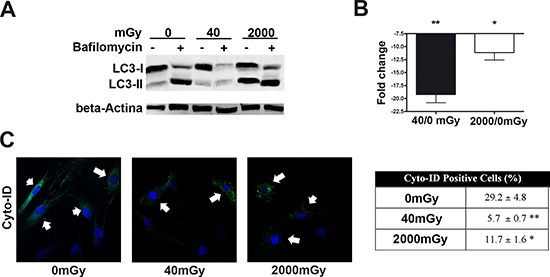
Autophagy detection assays Panels **(A, B)** – The picture shows western blot detection of LC3-I and LC3-II in irradiated and control MSC. Following irradiation (40 and 2000 mGy), cells were incubated for six hours and then harvested for western blot analysis. Two hours before the end of cell sample preparation, irradiated and control MSC cultures were incubated with 100 nM Bafilomycin A1 (inhibitor of lysosomal degradation) or PBS to detect autophagic flux. We used Gel Doc 2000 Gel Documentation Systems (Bio-Rad, CA, USA) to measure LC3-I and II band intensities that were normalized with beta-actin (chosen as loading control). We determined autophagic flux (AF) for LC3 II as follows: IR-treated MSC AF = (IR-treated MSC + Bafylomycin A1) - (IR treated MSC + PBS); Control MSCs AF = (Control MSC + Bafylomycin A1) - (Control MSC + PBS). Change in autophagic flux (ΔAF) between IR-treated and control MSC was calculated as ΔAF = IR-treated MSC AF - Control MSC AF. The graph shows AF changes in IR-treated MSC compared to control cultures. Data are expressed in change folds (*n* = 3; *p < 0.05). Panel **(C)** – Cyto-ID assay. Representative microscopic fields of cells with active autophagy (green) in irradiated and control cells are shown. Nuclei were counterstained with Hoechst 33342 (blue). Arrows indicate Cyto-ID-positive cells. The table shows mean percentage value of Cyto-ID-positive cells (± SD, *n* = 3, *p < 0.05, **p < 0.01).

Following IR treatment, we determined the level of LC3 isoforms, both in the presence and absence of bafilomycin A1, an inhibitor of lysosomial degradation. The amount of LC3 at a certain time point does not indicate autophagic flux, and therefore to determine changes in autophagic flux it is important to measure the amount of LC3-II in the presence and absence of lysosomal protease inhibitors. Both low and high doses of X-rays induced a reduction of autophagic flux, suggesting an impairment of the autophagy process (Fig. 3A, B).

Inhibition of autophagy in IR-treated cells was confirmed by Cyto-ID^®^ Autophagy Detection assay (Enzo Life Sciences). The assay measures autophagy with a cationic amphiphilic tracer dye that rapidly partitions into cells and enables labeling of vacuoles associated with the autophagy pathway. The percentage of cells expressing active autophagic vacuoles was significantly decreased in irradiated MSCs compared with the control (Fig. 3C).

### IR triggered DNA repair processes, but some foci of unrepaired DNA persisted

The increase in senescence phenomena induced by IR suggests that DNA damages were not completely repaired and that cells may have persistent foci of damaged DNA. Following genotoxic stress, the activation of DNA damage repair (DDR) pathway occurs during short period of time and may persist for some hours. For this reason we carried out all the DDR analyses at several time points: one, six and 48 post irradiation.

We evaluated the activation of the DNA repair system by looking at Ataxia telangiectasia mutated kinase (ATM), which is a kinase that regulates DNA repair. Activation of ATM by autophosphorylation on Ser1981 occurs in response to exposed DNA double-strand breaks [26]. ATM and its downstream effectors signal in pulses, thus following its binding to DNA-damage foci, ATM dephosphorylates and dissociates from the foci [27]. Activation of ATM was carried out together with determination of cycling cells by Ki67 immunostaining [28], used to evaluate the trigger of the DNA repair mechanism and cell cycle.

**Figure 4:**
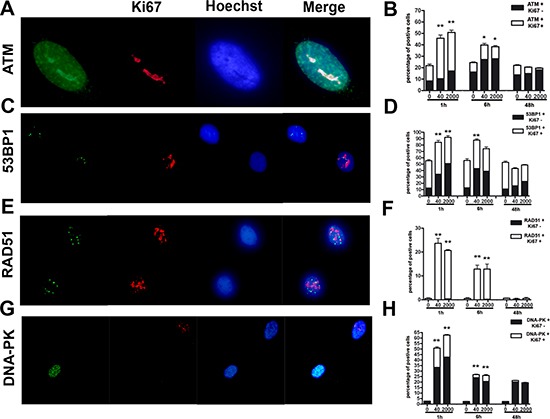
Evaluation of DNA damage and repair Panels **(A, B)** – ATM immunostaining. Fluorescence photomicrographs shows a typical cell stained with anti-ATM (green) and Ki67 (red). Nuclei were counterstained with Hoechst 33342 (blue). A representative microscopic field for each treatment is shown. The mean percentage of ATM- and Ki67-positive cells is indicated in the graph (± SD, *n* = 3, *p < 0.05). Panels **(C, D)** – 53BP1 immunostaining. Fluorescence photomicrographs show cells stained with anti-53BP1 (green) and Ki67 (red). Nuclei were counterstained with Hoechst 33342 (blue). A representative microscopic field for each treatment is shown. The mean percentage of 53BP1- and Ki67-positive cells is indicated in the graph (± SD, *n* = 3, *p < 0.05). Panels **(E, F)** – RAD51 immunostaining. Fluorescence photomicrographs show cells stained with anti-RAD51 (green) and Ki67 (red). Nuclei were counterstained with Hoechst 33342 (blue). A representative microscopic field for each treatment is shown. The mean percentage of RAD51- and Ki67-positive cells is indicated in the graph (± SD, *n* = 3, *p < 0.05). Panels **(G, H)** – DNA-PK immunostaining. Fluorescence photomicrographs show cells stained with anti-DNA-PK (green) and Ki67 (red). Nuclei were counterstained with Hoechst 33342 (blue). A representative microscopic field for each treatment is shown. The mean percentage of DNA-PK- and Ki67-positive cells is indicated in the graph (± SD, *n* = 3, *p < 0.05).

Both low and high dose irradiation produced a significant number of ATM-positive cells one and six hours post treatment, but 48 hours post-IR, the number of positive cells decreased compared to the level observed in control cultures (Fig. 4A, B). This trend was noticed both in cycling (Ki67+) and resting cells (Ki67-).

We then analyzed more in depth the double strand break repair (DSB) pathways by looking at the expression of 53BP1, involved in DSB pathway choice. We evaluated also the expression of RAD51 and DNA-PK, which have a key role in the activation of homologous (HR) and non-homologous-end-joining (NHEJ) DNA repair systems, respectively [29–31]. One and six hours post treatment, we evidenced a significant number of 53BP1-positive cells in both low and high dose irradiated, but 48 hours post-IR, the number of positive cells decreased compared to the level observed in control cultures (Fig. 4C, D). This trend was noticed both in cycling (Ki67+) and resting cells (Ki67-).

In control cells, we did not find any expression of RAD51. In the irradiated cells, the expression was evident almost exclusively in Ki67-positive cells, and only one and six hours post treatment; 48 hours post-IR, no staining was detected (Fig. 4E, F).

Increased number of DNA-PK-positive cells was detected one and six hours post treatment, in both low and high dose irradiated cultures. Then, 48 hours post-IR, the number of positive cells decreased to the level observed in control cultures (Fig. 4G, H). This trend was noticed both in cycling (Ki67+) and resting cells (Ki67-).

Altogether, these findings proved that DNA repair system was properly activated following IR, it remained to determine if all DNA damages were repaired.

An important downstream target of ATM is the histone H2AX, which is rapidly phosphorylated following activation of ATM. The phosphorylated H2AX (gamma-H2AX) is involved in the recruitment and/or retention of DNA repair and checkpoint proteins. Gamma-H2AX foci are marker of damaged DNA that is undergoing the repair process, and the persistence of these foci several hours (or days) following genotoxic stress is a sign of the presence of unrepaired DNA in cells' nuclei [32, 33].

**Figure 5:**
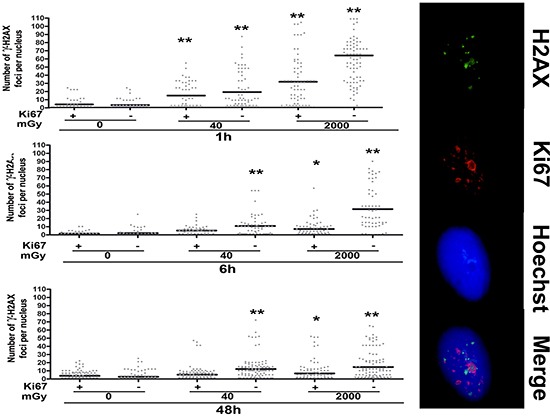
Gamma H2AX staining Fluorescence photomicrographs show the merging of cells stained with anti-H2AX (green), anti Ki-67 (red) and Hoechst 33342 (blue). A representative microscopic field for each treatment is shown. – Graph shows the degree of H2AX phosphorylation. This was evaluated by counting the number of gamma-H2AX immunofluorescent foci per cell. Foci number was determined for 200 cells. Each dot represents an individual cell. Black bars indicate mean value for each category (*n* = 3, *p < 0.05, **p < 0.01).

Low and high dose irradiation caused a significant augmentation of cells with a high number of gamma-H2AX foci that persisted even 48 hours post treatment (Fig. 5). Of interest, this was observed mainly in resting cells (Ki67-). This suggests that cycling cells may have a more effective DNA repair system (Fig. 5).

## DISCUSSION

Researchers and physicians agree that high dose radiation has deleterious effects in humans, including, but not exclusively, the triggering of neoplasia. The biological effects of low levels of radiation have been investigated for several years, but there is still no concordance on this issue among scientists. Understanding the risks of low radiation doses still has societal importance, and to have a clear-cut vision of the effects of low dose radiation on humans, larger epidemiological studies are required to quantify the risk to a useful degree of precision [34]. Moreover, the effects of low dose radiation should not be confined to cancer risk, as aging-related diseases may also be triggered by the impairment of homeostatic capacity of organs and tissues following exposure to low levels of radiation. In this setting, a key role is played by stem cells, which greatly contribute to tissue regeneration and are highly susceptible to progressive accumulation of DNA damage given their long half-life.

We focused our interest on the effects of very low doses of radiation on MSC, which contribute to the homeostasis of bone marrow and other organs and give rise to mesodermal derivatives.

Our *in vitro* data suggest that MSC functions were affected by low dose radiation exposure. *In vivo* experiments should be carried out to evaluate if MSC in their physiological environment may be more resistant to IR injury [35, 36]. Indeed, some preliminary reports suggested that bone marrow exposure to IR induced rapid depletion of hematopoietic stem cells (HSC) and of their progenitors, while MSC can survive radiation [35]. It remains to be determined if surviving MSC are senescent cells that cannot contribute to bone marrow homeostasis, including HSC self-renewal and differentiation.

The main consequence of low-level radiation exposure, besides reduction of cell cycling, is the triggering of senescence, while the contribution to apoptosis is marginal (Fig. 1 and 2). Of note, the increase in senescence is progressive from 40 to 2000 mGy (Tab. 1), and exposure to high dose radiation preferentially induced senescence rather than apoptosis. This could be a quite-specific property of MSC, since even at very high radiation doses (4 – 20 Gy), these cells enter senescence rather than apoptosis [5, 37]. The consequence of senescence is the loss of stem cell properties, as seen in the significant reduction of the cloning capacity of MSC cultures (CFU assay shown in Fig. 2). A recent report demonstrated that MSC may retain their defining stem cell features after exposure to high dose radiation (2 – 4 Gy) [38]. This may not contradict our findings, since senescence reduced the number of MSC clones, but the few remaining may retain their differentiation capacity.

Another issue that our study tried to address was the complex relationship that exists between senescence and autophagy. In some contexts, the induction of senescence is dependent on a prior induction of autophagy. In contrast, several reports have shown that the inhibition of autophagy promotes senescence. The explanation for these two opposite outcomes may rely on the fact that in some experimental conditions, cells try to cope with exogenous or endogenous stress by activating autophagy that eliminates damaged components. In this scenario, autophagy protects from senescence and/or apoptosis, and its inhibition may trigger these two events. On the other hand, if autophagy cannot cope with stress-induced damage, it triggers apoptosis or senescence as the final cellular reaction to stress [23, 24].

Our results suggest that, in our experimental conditions, the autophagy counteracts deteriorative processes, and its decline triggers senescence along with a decrease in stemness. Our data are in agreement with the finding of Hou et al., showing that autophagy prevents irradiation injury of MSC [39]. Of interest, Hou et al. carried out a study only on high IR (6000 Gy). We, for the first time, showed that even low IR may greatly injury MSC. Impairment of autophagy and trigger of senescence following radiation concords with studies showing that inhibition of mTOR promotes autophagy and may rescue cells from senescence [40, 41]. Further studies could exploit the blockage of mTOR pathways as a therapeutic target for patients undergoing IR treatment. Indeed, it has been demonstrated that rapamycin, a mTOR inhibitor, enhances long-term hematopoietic reconstitution of mouse hematopoietic stem cells by inhibiting senescence [42, 43].

The increase in ATM staining six hours post-irradiation and its drop to basal level at 48 hours, along with an enduring gamma-H2AX staining, suggest that MSC properly activated the DNA repair signaling system, but some damages persisted unrepaired. Indeed, ATM and its downstream effectors signal in pulses that arise from periodic examinations of the status of the DNA damage. Several phosphatases fine-tune ATM activity in order to prevent illegitimate activation of the DNA damage response in the absence of damage, as well as to allow rapid cessation of the signal once DNA damage sites have been recognized, irrespective of whether they have been properly repaired. On the other hand, gamma-H2AX can remain bound to unrepaired DNA, as suggested by the kinetics analysis of gamma-H2AX clearance after IR or other DNA-damaging agents [32, 33].

The existence of persistent unrepaired DNA foci may be the trigger of senescence phenomena as already evidenced by Campisi's team, who showed that persistent foci of damaged DNA, termed DNA-SCARS, sustain damage-induced senescence growth arrest [44].

Endurance of gamma-H2AX foci is mainly observed in non-cycling cells (Ki67-), indicating that the impaired DNA repair capacity of irradiated MSC seems mainly related to reduced activity of the NHEJ system, rather than the HR. Indeed, the NHEJ is the only double-strand break repair system that is active in non-cycling cells.

In conclusion, our data showed that human bone marrow MSC are sensitive to very low levels of radiation and trigger senescence due to impaired autophagy and DNA repair capacity. The presence of senescence rather than apoptosis may be more dangerous for the proper functioning of bone marrow. Apoptotic cells are quickly cleared by phagocytes and may be replaced by new cells [45]. Senescent cells may persist for longer periods in tissues and can affect neighboring cells by secreting a plethora of molecules. The use of secreted molecules for senescence can have potential positive and negative effects on a body's health. Factors secreted by senescent cells could constitute a danger signal that sensitizes normal neighboring cells to senesce. This may reduce the possibility of damaged cells at risk of neoplastic transformation failing to enter senescence. There are also potential negative consequences of the presence of senescent cells in a tissue. A few senescent cells may sensitize many neighboring healthy cells to senesce with the accumulation of a huge number of senescent cells that impair tissue function and contribute to organismal aging. Moreover, in later stages of tumorigenesis, tumor cells can misuse secreted factors for their growth instead of stopping their proliferation

On this premise, even if some reports have shown that HSC appear to be resistant to low dose radiation effects, the senescence of MSC induced by low-level IR may indirectly affect HSC functioning and may also have wider repercussions on bodily health given the huge number of cytokines, growth factors and immune system modulators that are produced by MSC [46].

Further studies on the *in vivo* effects of low dose radiation on MSC functioning are highly enticing.

## MATERIALS AND METHODS

### MSC cultures

Bone marrow was obtained from healthy donors (age 18–40 years) after informed consent. We used plastic adherence and minimal criteria suggested by International Society for Cellular Therapy to identify mesenchymal stromal cells (MSC), which contain a subpopulation of stem cells [47].

We separated cells on a Ficoll density gradient (GE Healthcare, Italy), and the mononuclear cell fraction was collected and washed in PBS. We seeded 1-2.5 × 10^5^ cells/cm^2^ in alpha-MEM containing 10% FBS and bFGF. After 72 hours, non-adherent cells were discarded, and adherent cells were further cultivated to confluency. Cells were then further propagated for the assays reported below. All cell culture reagents were obtained from Euroclone Life Sciences (Italy).

### Irradiation

Exponentially growing cells (passage 3–4) were irradiated with 40, 160, 640 and 2000 mGy X-ray at room temperature. X-rays were administered via a Mevatron machine (Siemens, Italy) operating at 6 MeV. Following irradiation, cells were further cultivated for six and 48 hours.

### Cell cycle analysis and cell proliferation assay

For each assay, cells were collected and fixed in 70% ethanol, followed by PBS washes, and finally were dissolved in a hypotonic buffer containing propidium iodide. Samples were acquired on a Guava EasyCyte flow cytometer (Merck Millipore, Italy) and analyzed with a standard procedure using EasyCyte software.

Cell proliferation was determined with the Quick Cell Proliferation Assay Kit II (Biovision, CA, USA). The assay is based on the cleavage of the tetrazolium salt to formazan by cellular mitochondrial dehydrogenase. The amount of the dye generated by activity of dehydrogenase is directly proportional to the number of living cells. The formazan dye produced by viable cells can be quantified by multi-well spectrophotometer by measuring the absorbance of the dye solution at 440 nm. We plated 1000 cells in 96-multiwell chambers and performed irradiation treatments. Cell proliferation was evaluated 1, 3 and 5 days post-irradiation.

### Nexin V assay

Apoptotic cells were detected using a fluorescein-conjugated Annexin V kit on a Guava EasyCyte flow cytometer, following the manufacturer's instructions.

### In situ senescence-associated beta-galactosidase assay

The percentage of senescent cells was calculated by the number of blue, beta-galactosidase-positive cells out of at least 500 cells in different microscope fields, as already reported [48].

### Colony Forming Unit (CFU) assay

MSC cultures were obtained from bone marrow as described above. Cultures were expanded to 70–80% confluency. On these cells (passage 0), we carried out CFU assay as reported [49]. In brief, 1000 cells were plated in 100 mm plates and were incubated for 15 days without medium change. The plates were collected, fixed and stained with 0.5% crystal violet. Stained colonies were identified under light microscope and were counted.

### Immunocytochemistry for detection of ATM, gamma-H2AX, Ki67, RAD51, DNA-PK and 53BP1

ATM (ab36810, ABCAM, UK), gamma-H2AX (2577, Cell Signaling, MA, USA), Ki67 (sc7846, SantaCruz Biotech, CA, USA), RAD51 (ab88572, ABCAM, UK) DNA-PK (sc390698, SantaCruz Biotech, CA, USA) and 53BP1 (ab172580, ABCAM, UK) were detected according to manufacturers' protocols. Hoechst 33342 staining was performed, and then cells were observed through a fluorescence microscope (Leica Italia, Italy). The percentage of ATM-, gamma-H2AX-, Ki67-, RAD51-, DNA-PK and 53BP1-positive cells was calculated by counting at least 500 cells in different microscope fields.

### CytoID autophagy detection kit

The Cyto-ID^®^ Autophagy Detection Kit (Enzo Life Science, NY, USA) measures autophagic vacuoles and monitors autophagic flux in live cells using a cationic amphiphilic dye that selectively labels autophagic vacuoles. We determined the percentage of Cyto-ID-positive cells according manufacturer's instructions.

### Western blotting

Cells were lysed in a buffer containing 0.1% Triton for 30 minutes at 4°C. 10–40 μg of each lysate was electrophoresed in a polyacrylamide gel, and electroblotted onto a nitrocellulose membrane. All the primary antibodies were used according to the manufacturers' instructions. Immunoreactive signals were detected with a horseradish peroxidase–conjugated secondary antibody (SantaCruz, CA, USA) and reacted with ECL plus reagent (GE Healthcare, Italy).

### Statistical analysis

Statistical significance was evaluated using ANOVA analysis followed by Student's t and Bonferroni's tests. We used mixed-model variance analysis for data with continuous outcomes. All data were analyzed with a GraphPad Prism version 5.01 statistical software package (GraphPad, CA, USA).

## SUPPLEMENTARY FIGURE


