# Supplementary material for: Low dose radiation induced senescence of human mesenchymal stromal cells and impaired the autophagy process
Source: Oncotarget. 2014 Dec 16;6(10):8155–66. doi: 10.18632/oncotarget.2692 (PMC4480742; doi:10.18632/oncotarget.2692)
Supplement: Supplementary file 1 [file oncotarget-06-8155-s001.pdf]

SUPPLEMENTARY FIGURE

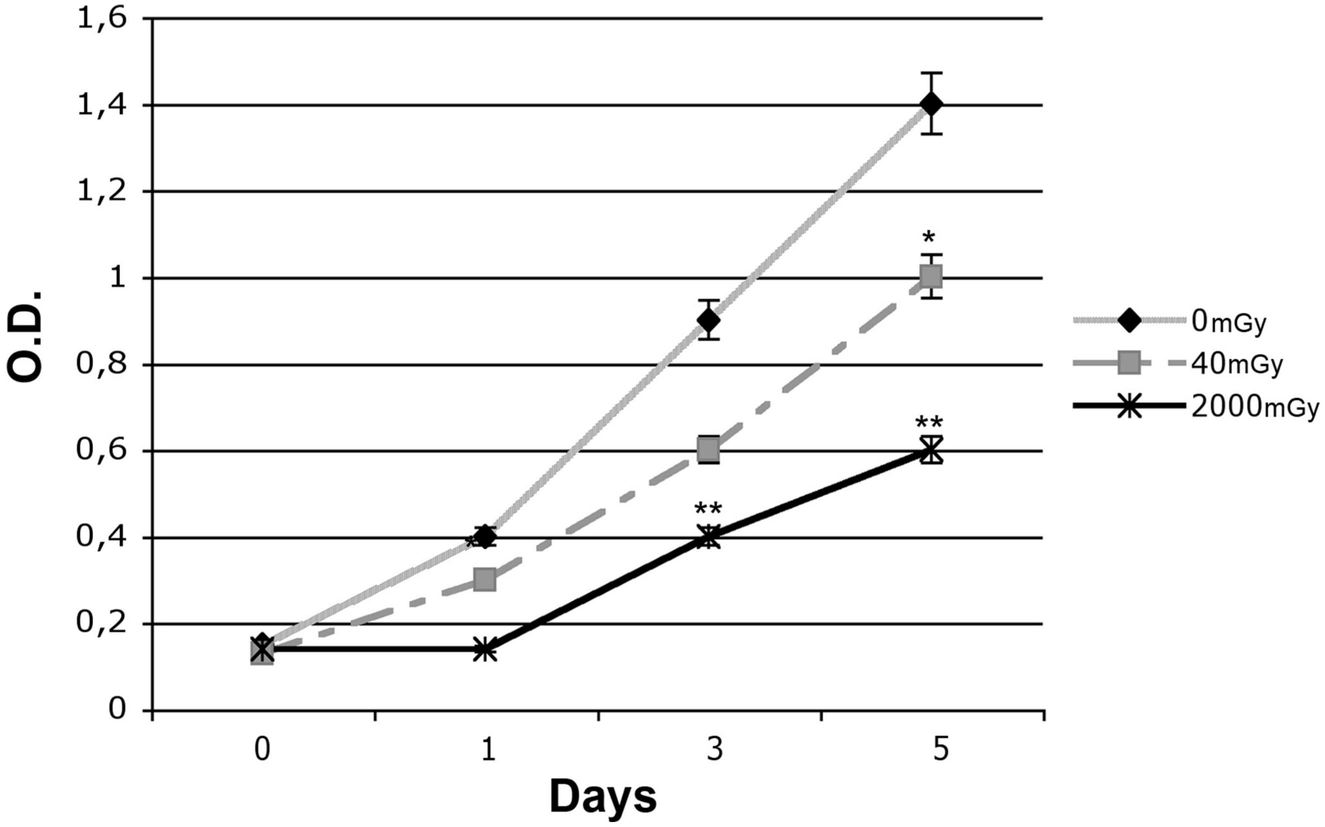

Supplementary Figure S1: Graphic shows MSC proliferation following X-ray treatment. The formazan dye produced by viable cells was determined by measuring the absorbance of the dye solution at 440 nm (Y-axis). 1,000 cells were plated in 96-multiwell chambers and irradiation treatments were perfomed. Cell proliferation was evaluated 1, 3 and 5 days post-irradiation. Data are expressed with standard deviation ($n = 3$, *$p < 0.05$, **$p < 0.01$).
